# Thermal and optical control of electronic states in a single layer of switchable paramagnetic molecules†
†Electronic supplementary information (ESI) available: Experimental section, electronic absorption spectra, additional magnetic data, complete ToF-SIMS and XPS analysis of **1–3** complexes, full set of XAS spectra with fitting curves of **2** and additional experimental details. See DOI: 10.1039/c5sc00163c



**DOI:** 10.1039/c5sc00163c

**Published:** 2015-02-12

**Authors:** Giordano Poneti, Lorenzo Poggini, Matteo Mannini, Brunetto Cortigiani, Lorenzo Sorace, Edwige Otero, Philippe Sainctavit, Agnese Magnani, Roberta Sessoli, Andrea Dei

**Affiliations:** a University of Florence , Department of Chemistry "Ugo Schiff" and INSTM Research Unit of Florence , via della Lastruccia 3-13 , 50019 Sesto Fiorentino , Italy . Email: giordano.poneti@unifi.it ; Email: matteo.mannini@unifi.it ; Fax: +39 055 4574913 ; Tel: +39 055 4573269; b “Guglielmo Marconi” University , Department of Applied Science and Technology , via Plinio 44 , 00193 Roma , Italy; c Synchrotron SOLEIL , BP 48 91192 Gif-Sur-Yvette , France; d Institut de Minéralogie , de Physique des Matériaux et de Cosmochimie , UMR7590 CNRS , Université Pierre et Marie Curie (Paris 6) , 4 place Jussieu , 75252 Paris , France; e University of Siena , Department of Biotechnologies , Chemistry and Pharmacy , INSTM Research Unit of Siena , Via A. Moro 2 , 53100 Siena , Italy

## Abstract

The control of the charge distribution in a monolayer of switchable cobalt–dioxolene complexes undergoing Valence Tautomerism (VT) has been achieved by means of thermal and optical stimuli. Thiol-derivatised VT molecules have been grafted on polycrystalline gold surface as monolayers from solution. X-ray photoelectron spectroscopy and time-of-flight secondary ions mass spectrometry evidenced the formation of a covalent bond between intact VT molecules and the surface and excluded the presence of physisorbed molecules. X-ray absorption spectroscopy revealed that the temperature- and light-induced conversion profiles of the monolayer closely reproduce the ones found for the crystalline phase. This study demonstrates that a wet chemistry based approach allows to transfer switchable paramagnetic molecules at the nanoscale, widening the playground to develop new hybrid molecular based architectures for novel technologies.

## Abstract 2

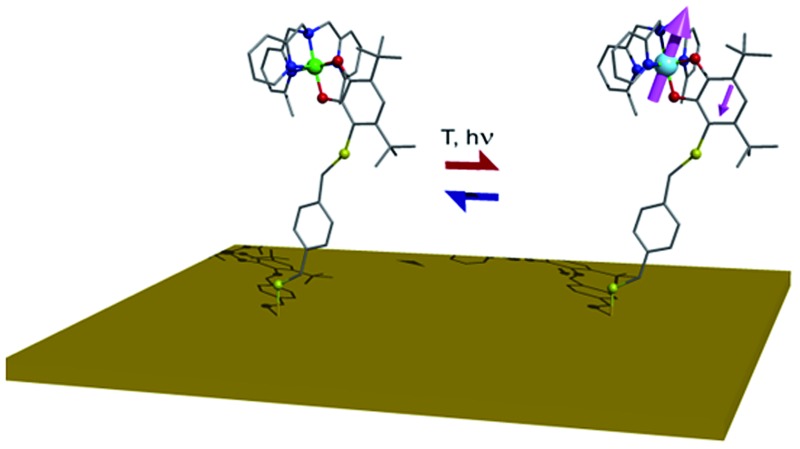
Thermally and optically induced Valence Tautomeric interconversion has been observed for a monolayer of a cobalt–dioxolene complex on gold.

## Introduction

Reversible conversion between two electronic states of bistable coordination complexes at the single molecule level promises breakthrough outcomes for information storage and processing technologies.^1^ In particular, the control of length and direction of the spin in paramagnetic switchable molecules represents a key feature to be used in quantum computation and molecular spintronic applications.^2–5^ The retention of switchability in molecular-inorganic architectures obtained by the regular assembling of molecules on conductive surfaces is a mandatory step towards their integration in hybrid devices. The possibility of obtaining bistable nano-assemblies as thin films and nanoparticles comprising Fe(ii)-based spin-crossover (SCO) materials, where switchability is due to an externally controllable spin state, has been suggested by several recent reports.^6–15^ These studies, however, highlighted that the direct molecular–substrate interaction can significantly modify the thermodynamics of the SCO equilibrium. While an HOPG (highly oriented pyrolytic graphite) supported SCO system has been reported to preserve its bulk phase switchability features,^16^ other sub-monolayer deposits of several Fe(ii) complexes show the “pinning” of their spin states – and loss of their original SCO behaviour – when evaporated on metallic surfaces.^8–14^

**Scheme 1:**
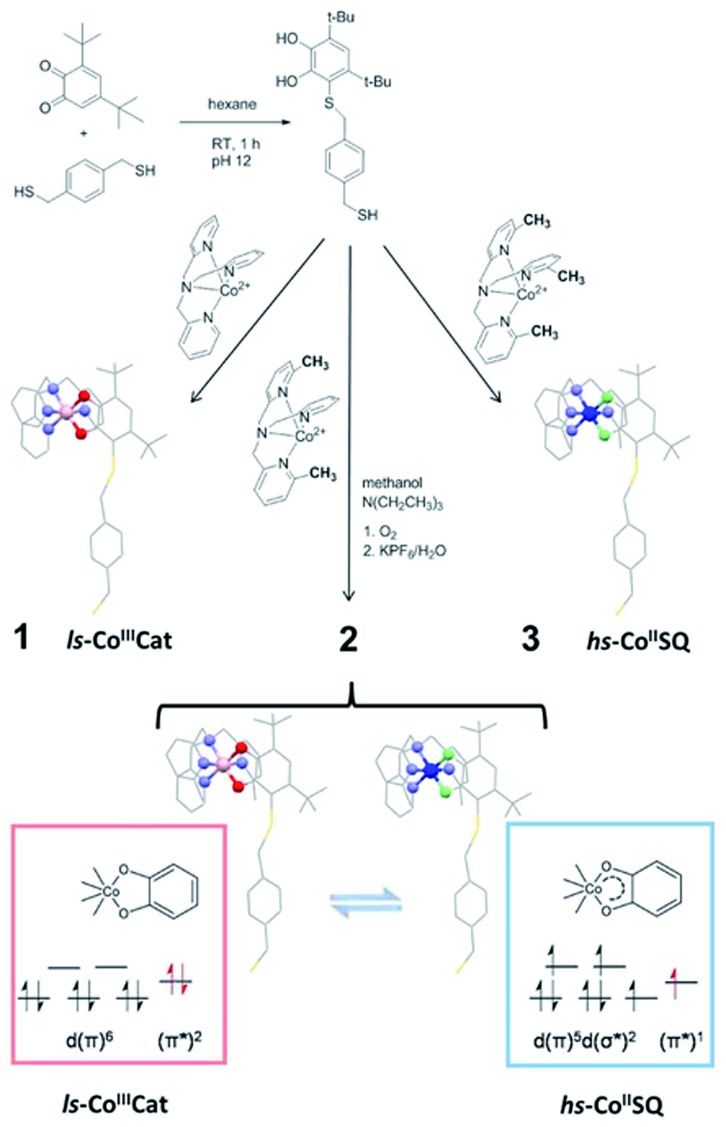
Synthetic pathway to the thiol-functionalised DBCatSH ligand and to the **1–3** complexes used in this study, highlighting their different charge distributions.

Promising alternative switchable systems for nanoscale investigation are those exhibiting a thermally or optically induced intramolecular charge transfer between an acceptor and a donor unit.^17^ This class of materials includes cyano-metallates^18–21^ and metal complexes showing Valence Tautomerism (VT from now on), an interconversion between redox isomers caused by electron transfer between the metal ion and an organic ligand.^22–24^ In order to investigate the VT behaviour on a metallic surface, we focused our interest on cobalt–dioxolene complexes,^25^ which can be considered as class II mixed valence systems, according to Robin and Day classification.^26^ These coordination compounds are the simplest and most widespread molecular systems displaying VT and rely on a [CoL*diox*] core, L being a tetradentate N-donating ancillary ligand and *diox* a chelating ligand belonging to the *ortho*-quinone (dioxolene) family. Proper chemical tuning of the reduction potentials of Co^3+/2+^ couple allows to prepare structurally related systems with different charge distributions, either ls-Co^III^Cat (ls = low-spin, Cat = catecholato), hs-Co^II^SQ (hs = high-spin, SQ = semiquinonato radical), or exhibiting VT.^27^ In the latter case, at low temperatures the diamagnetic ls-Co^III^Cat redox isomer is the ground state, but entropy driven intramolecular electron transfer triggers the reversible formation of the paramagnetic hs-Co^II^SQ species upon heating the system (Scheme 1).^25^ At cryogenic temperatures the same interconversion can be induced by visible^17^ and soft X-ray irradiation;^28^ once the stimulating source is removed, a slow decay to the ls-Co^III^Cat ground state occurs with a temperature dependent characteristic time.^29^ DFT calculations have recently proposed that switching can be induced by the application of an electric field, suggesting that detection and modulation of the magnetic state of these complexes can be obtained at the single molecule level by using Scanning Tunnel Microscopy-based techniques.^30^

Whatever the external stimulus chosen to control the charge distribution, the perspective of integration of these molecules in spintronic devices obviously requires the development of a reliable method to obtain a monolayer of VT complexes which retains their switchable properties. As a first step in this direction we have recently shown that a sulfur-based functionalisation can be introduced in a [CoL*diox*] unit maintaining the switchability of the core,^31^ thus opening the way to a wet-chemistry approach for the deposition of VTs on noble metal surfaces. This technique^32^ has afforded successful examples of bidimensional structuration for different classes of molecular materials, including molecular nanomagnets,^33^ redox switches based on organic radicals,^34^ and organic photochromic materials.^35^ In the case of charge transfer systems, to the best of our knowledge, self-assembling from solution has not yet been carried out.

This work reports on the deposition on a metallic surface of a monolayer of a charge–transfer based molecular switch. Functionalisation of the molecular core with a thiol-bearing moiety allowed the chemisorption through self-assembly from a diluted solution. A multi-technique (X-ray photoelectron spectroscopy, XPS, and time-of-flight secondary ion mass spectrometry, ToF-SIMS) study proved the formation of a single layer of intact molecules on top of polycrystalline gold surface. Moreover, XPS and X-ray absorption spectroscopy (XAS) analysis provided the experimental proof of the retention of both entropy driven and light induced interconversion of molecules grafted to the gold surface.

## Results and discussion

In order to graft [CoL*diox*] systems on a gold surface, we prepared a family of complexes of general formula [Co(Me_*n*_tpa)DBCatSH](PF_6_)·CH_3_OH (*n* = 0, 2, 3 for compound **1**, **2** and **3**, respectively), where Me_*n*_tpa are differently methylated derivatives of tris-pyridil-amine and DBCatSH is the 3,5-di-*tert*-butyl-catecolate ligand functionalised with a thiol moiety (Scheme 1).

**Fig. 1:**
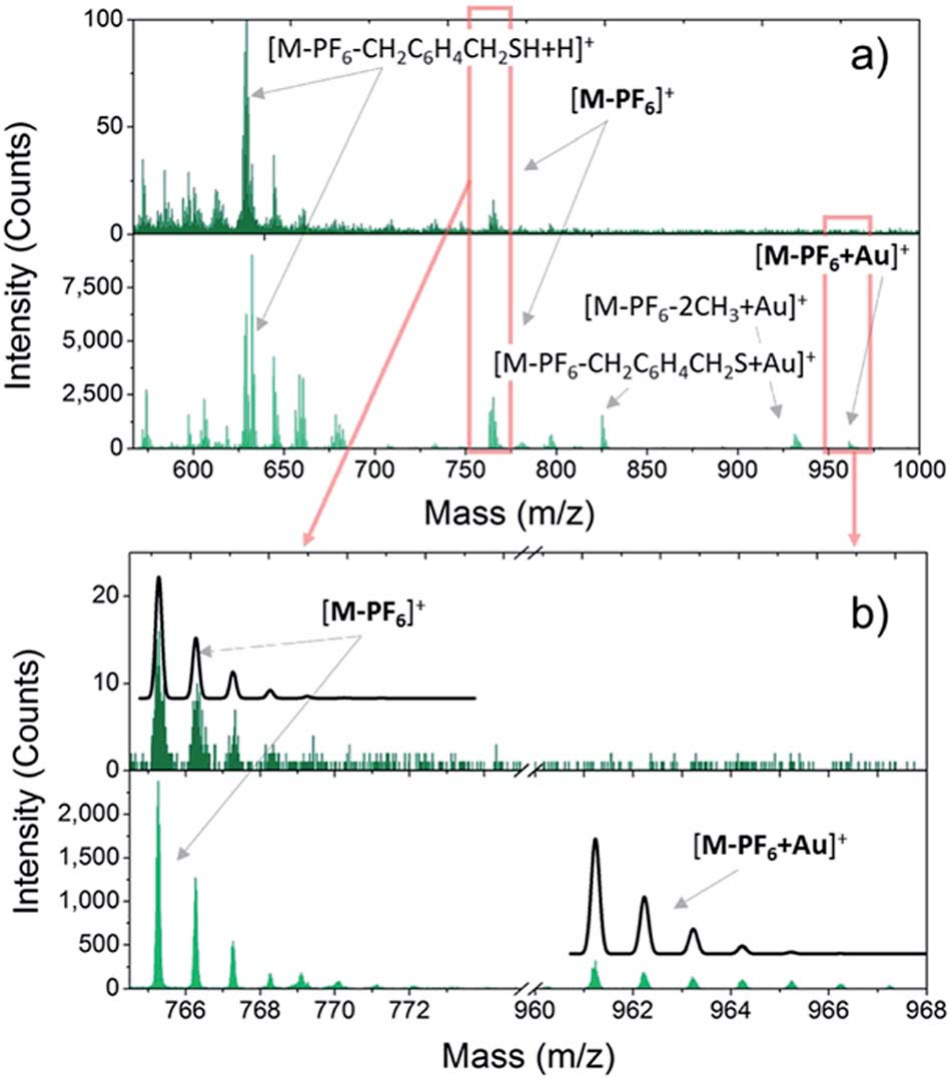
a): Positive ToF-SIMS spectra of bulk (dark green sticks) and monolayer (green sticks) of **2** measured in static regime. (b): Magnification of the [M-PF_6_]^+^ and [M-PF_6_-H + Au]^+^ regions (enclosed in the red rectangles in a) of bulk and monolayer samples. The calculated isotopic distribution pattern expected for each fragment is reported as a black line.

Electronic (UV-Vis) spectroscopy, elemental analysis and mass spectrometry (Fig. 1 and S1†) indicate that the complexes share the molecular structure reported in Scheme 1 differing only for the number of methyl groups present in the ancillary ligand. UV-Vis, magnetometry and XPS data (Fig. S1, S2 and S3†) evidence a room temperature charge distribution of ls-Co^III^Cat for **1**, hs-Co^II^SQ for **3**, and a mixture of the two for **2**, as expected on the basis of previous reports on other [CoL*diox*] complexes featuring the same ancillary ligands.^27,31,36,37^ The temperature dependence of the molar magnetic susceptibility *χ*_M_ *T* occurring in **2** (Fig. S2†) points out a reversible interconversion between the two redox isomers in the solid state, in line with an entropy driven VT process. Using the *χ*_M_ *T* values of **1** and **3** as references for ls-Co^III^Cat and hs-Co^II^SQ phases (eqn S(1)†), it is possible to quantitatively describe the thermal distribution profile of **2**. The VT interconversion has a very gradual character: at low temperatures it displays a remaining 40% hs-Co^II^SQ fraction that reaches 66% at 300 K, occurring in a temperature range broader than the experimentally accessible one in our setup. Broad thermally induced electronic transitions in switchable molecular materials are usually related to the absence of intermolecular interactions in the solid state,^38,39^ and have been previously found for other sulfur-functionalised [CoL*diox*] switchable complexes.^31,40^ The VT conversion of **2** can also be triggered at low temperature by light (Fig. S4†): excitation of the ligand to metal charge–transfer band at 10 K turns 15% of the ls-Co^III^Cat content to the hs-Co^II^SQ metastable phase. Upon heating at a 0.3 K min^–1^ rate, the initial state is recovered at 75 K, while the temperature with the highest measurable relaxation rate (*T*_LIESST_) is 55 K. Compared with previously analyzed [CoL*diox*] VT systems,^28,29,31^ these data confirm that light-triggered bistability is a strictly molecular phenomenon, suggesting a possible exploitation at the nanoscale.

Once the VT behaviour of **2** in the solid state has been confirmed, we prepared monolayers of **1–3** complexes by self-assembly from solution and checked the integrity of the surface supported molecules by ToF-SIMS and XPS. Fig. 1 compares the positive ions mass spectra of bulk and monolayer samples of **2** (for complete peak assignation see Table S1†). The comparison among the spectrum of **2** with those of **1** and **3** (Fig. S5†) points out a clear correlation in the fragmentation patterns, which arises from the different number of methyl groups in the family, and evidences that the DBCatSH ligand is the main site of molecular fragmentation. The presence of intact [Co(Me_*n*_tpa)DBCatSH]^+^ cations on surface is witnessed by the observation of the corresponding [M-PF_6_]^+^ peak in each spectrum (737.22 *m*/*z*, 765.25 *m*/*z* and 779.31 *m*/*z* for **1**, **2** and **3**, respectively). Moreover, support to the formation of a single layer of molecules on surface is given by the presence of the molecular cation peak bound to a gold atom, [M-PF_6_-H + Au]^+^, absent in bulk samples. This suggests the formation of molecule–substrate covalent bond, in analogy to what has been observed on other systems assembled on Au(111).^41^

**Fig. 2:**
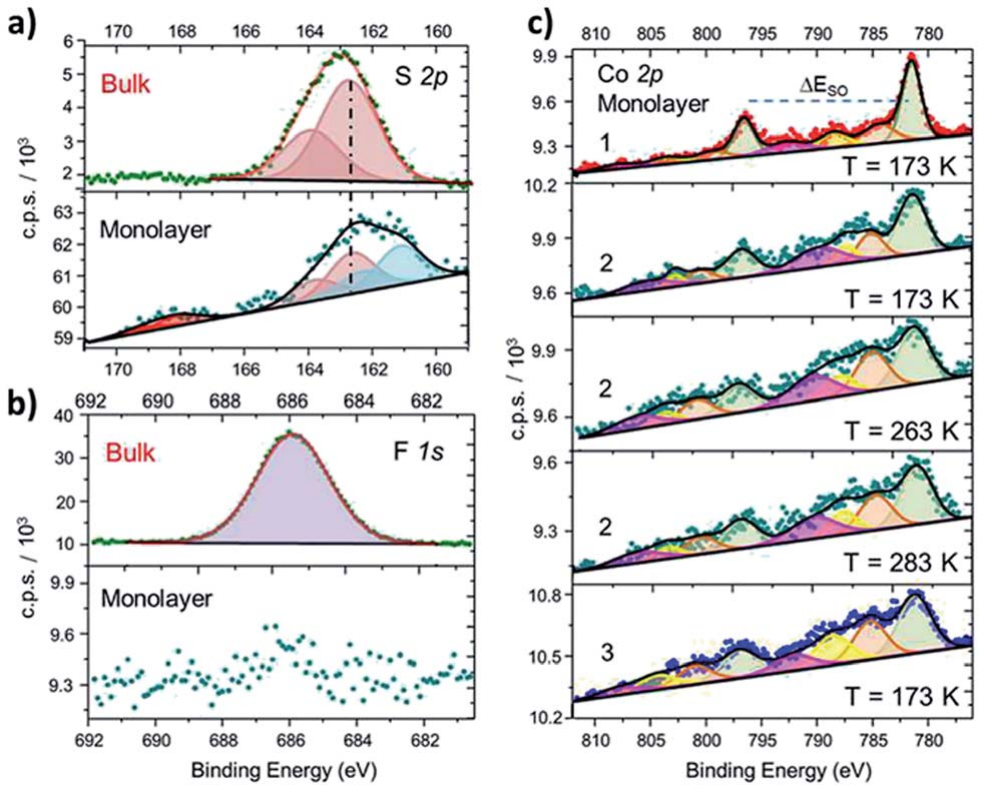
(a): Comparison between S2p XPS spectra of **2** in the bulk phase and as a monolayer, along with deconvolution of the photopeaks and corresponding best fitting lines (similar results for **1** and **3** and fitting details are in the ESI†). (b): Disappearance of the F1s photopeak, observed in bulk phase of **2**, when measuring a monolayer sample of the same derivative. (c): Comparison of XPS Co2p spectra of monolayers of **1**, **2** (taken at three different temperatures) and **3**, along with corresponding deconvoluted photopeaks and best fitting lines.

In order to further investigate structural and electronic properties of surface supported molecules, an XPS investigation has been performed on monolayer and bulk samples of **1–3** complexes. The direct comparison of the S2p regions in the solid state and monolayer of **2** (Fig. 2a) proves the chemisorption on Au (similar results obtained for **1** and **3** are reported in Fig. S6†). In the bulk phase, the S2p region can be reproduced using a photoemission peak centered at 162.7 eV, with a spin–orbit splitting (Δ*E*_SO_) of 1.2 eV, as expected for the very similar chemical environment of the two sulfur atoms.^42^ In the monolayer sample, an additional signal appears at 161.7 eV, integrating 46% of the overall S2p photopeak area: this component is directly attributed to the thiol group covalently bound to the Au surface, in analogy with previous reports on thiol based monolayers.^43,44^ A minor (8%) fraction at 168.4 eV is assigned to spurious oxidation of the sulfur atoms, usually related to defects in the thiol-based molecular packing on the surface.^41^

The semiquantitative analysis and the stoichiometric ratios (Table 1 and Fig. S7†) are in accordance with the calculated and ToF-SIMS data, suggesting that the majority of surface supported molecules retains the [CoL*diox*] cation structure found in the solid state. Moreover, the absence of counter-anion for the surface-supported molecules, monitored by the disappearance of F1s photopeak in monolayer samples (Fig. 2b), is an experimental evidence of the absence of physisorbed molecules on the Au(111) surface.

**Table 1:** Theoretical and XPS estimated atomic percentages (relative error about 5%) and ratios for **1–3** complexes in bulk and monolayer environments

Bulk	Co 2p	N 1s		S 2p		P 2p		F 1s	
	%	%	N/Co	%	S/Co	%	P/Co	%	F/Co
1	6.7	29	4.3	14.0	2.1	7.6	1.1	42	6.3
2	6.7	26	4.0	13.7	2.0	7.3	1.1	45	6.6
3	7.6	27	3.7	11.7	1.5	6.9	0.9	45	6.0
Theor.	7.1	28.6	4.0	14.3	2.0	7.1	1.0	42.9	6.0

Mono-layer	Co 2p	N 1s		S 2p		P 2p		F 1s	
	%	%	N/Co	%	S/Co	%		%	
1	14.8	53	3.6	31	2.1	—		—	
2	14.9	54	3.7	30	2.0	—		—	
3	14.0	53	3.8	32	2.3	—		—	
Theor.	14.3	57.1	4	28.6	2	—		—	

Besides providing stoichiometric information, Co2p XPS spectra yield clear fingerprints of charge distributions in cobalt–dioxolene adducts,^31^ and have been thus acquired for monolayer samples of **1–3** to analyze the effects of surface deposition on the electronic state of the metal ion (Fig. 2b). The Co2p spectrum of a monolayer of **1** has the typical lineshape of ls-Co^III^ photoemission:^31,45,46^ it shows a main peak at 781.5 eV, integrating about 50% of the overall signal area, and minor satellites at 784.7, 788.0 and 792.2 eV, with Δ*E*_SO_ contributions separated by 15.1 eV, in analogy to its bulk analogue (Fig. S3†). Complex **3**, on the other hand, presents a more structured spectrum, with high intensity satellites, as expected for 3d ions with unquenched orbital momentum like hs-Co^II^ in distorted octahedral environment. The main peak shifts to 781.2 eV and integrates about 40% of the whole signal area, with satellites at 785.2, 788.6 and 792.1 eV, and a Δ*E*_SO_ of 16.0 eV, in accordance with results obtained for bulk phase (Table S2†) and literature data.^31,46^ The Co2p photoemission of both samples does not display any temperature dependence in the 170–300 K range. Thus, surface deposition did not affect the electronic ground states of these complexes, as expected because of the large energy difference between the two possible redox isomers in both **1** and **3**. The room temperature XPS Co2p spectral features of a monolayer of **2** are different from those reported above: even displaying a closer resemblance with the latter, **2** shows a slightly lower Δ*E*_SO_ (15.8 eV), suggesting the coexistence in the sample of both charge distributions. Moreover, on cooling from 283 K to 173 K, an increase in the main peak contribution to the overall signal intensity (from 40% up to about 46%, respectively) parallels a reduction in the Δ*E*_SO_ (15.8 to 15.4 eV), indicating the presence of a thermally driven VT conversion in the monolayer.

**Fig. 3:**
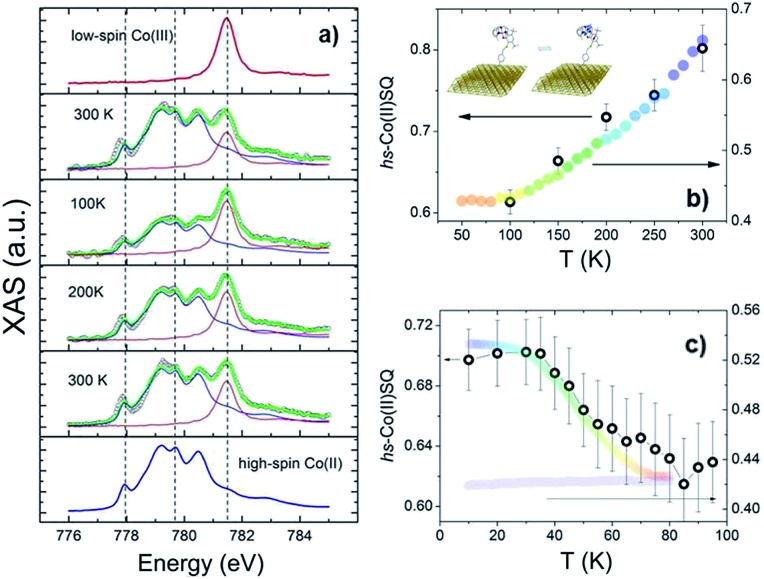
(a) Temperature evolution of the normalised Co L_3_ edge XAS spectra of a monolayer of **2** (empty black dots) along with high-spin Co^II^ and low-spin Co^III^ spectra (blue and red line, respectively) used as reference signals for the spectral deconvolution (green lines). Broken lines are guides to the eye. (b and c) high-spin Co^II^ thermal distribution profile (empty circles) obtained from XAS spectra taken before (b) and after (c) laser light irradiation. Massive phase rescaled data are reported as wide coloured bands for comparison.

To investigate VT equilibrium at the nanoscale with higher accuracy, XAS has been used. Synchrotron-based absorption techniques are unmatched tools to analyze submonolayer deposits of bistable molecular systems, providing the requested sensitivity to monitor oxidation^47,48^ as well as spin states^9,13,14^ and molecular orientation^49^ on surfaces. Fig. 3 displays the temperature evolution of the Co L_3_ edge absorption spectrum of a monolayer of **2** in the 100–300 K range (for the corresponding L_2_ edges spectra see Fig. S8,† while data taken at additional temperatures are reported in Fig. S9†). The 300 K spectrum confirms the coexistence of ls-Co^III^Cat and hs-Co^II^SQ redox isomeric forms in the monolayer, closely resembling the bulk phase behaviour. Isothermal time dependence of the Co L_2,3_ XAS spectra acquired on the same spot of the sample (see Fig. S10†) discards X-ray irreversible alterations of the electronic structure of the grafted molecules. The observed edge-jump (Fig. S11†), moreover, corresponds to about 10% of what is observed in the bulk samples of structurally related [CoL*diox*] VT systems taken in similar experimental conditions.^28,31^ Considering that only the first few nm of a bulk sample are investigated in this experiment, the much weaker signal detected for the film provides an additional proof of the monolayer thickness of the studied deposit.^41^ Upon cooling from 300 to 100 K the spectral features related to the ls-Co^III^ isomer gain intensity while the signal coming from the hs-Co^II^ one weakens; moreover, heating the sample back to 300 K the initial spectral lineshape is restored.

These experimental evidences are in line with the occurrence of a reversible entropy driven VT interconversion in the monolayer. In order to quantitatively analyse the hs-Co^II^ thermal distribution profile, the experimental Co L_3_ spectra have been fitted using the experimental spectra of reference complexes^31^ for the limiting redox isomeric distributions ls-Co^III^Cat and hs-Co^II^SQ (for fitting details see the ESI†). The results, displayed in Fig. 3, evidence a VT conversion that is comparable with that observed by traditional magnetometry for the bulk phase, the major difference being the 20(2)% increase in the remaining hs-Co^II^SQ molar fraction at low temperature found for the monolayer. This feature has been often documented for entropy driven interconversions of bistable molecular nano-assemblies, where the ratio between surface and bulk molecules becomes physically relevant, like thin films and nanoparticles of SCO systems.^7^ The reduction of intermolecular elastic interactions for surface-supported molecules, leading to lowered enthalpic and entropic changes accompanying the thermally driven SCO equilibrium, is the generally accepted explanation for the presence of an unconverting fraction at low temperature.^39^ For electron transfer processes, a similar behaviour has been theoretically described^50^ and experimentally observed for solid solutions of VT systems,^51^ and is thus extendable to our case, where we move from a 3D network of elastic interactions (solid state) to a monolayer.

Our analysis points out that the employed chemical grafting protocol has no significant effect on the charge distribution of **1–3** complexes and in particular that it is possible to quantitatively retain the switchability of **2** at the monolayer level. The preservation of ls-Co^III^Cat and hs-Co^II^SQ charge distributions for monolayers of **1** and **3** complexes, respectively, is not surprising, once evidenced their intact deposition on the Au(111) surface. Indeed, the charge distributions of similar systems have been shown to be scarcely affected by environmental effects in bulk phases.^48,52^ The experimental evidence of the entropy driven VT interconversion of **2** at the monolayer level, on the other hand, could have not been safely *a priori* predicted. In fact, if thermally induced VT processes have been observed in the past in diluted phases (like glass,^53^ solutions,^22,54–56^ polymeric dispersions,^57^ nanoparticles^58,59^), they have also been shown to be strongly dependent on the crystal packing in the solid state.^60,61^ In particular, transition temperatures of [CoL*diox*] systems are known to depend on the available volume per molecule in the lattice and display large changes as a function of the nature of the counter-ions^62^ and the crystallisation solvent.^29,52^ By employing a VT system displaying a gradual transition, we have demonstrated that entropy driven equilibria can be safely reproduced even on single molecular layers on top of metal surfaces.

The last issue of our analysis concerned the capability to photo-induce the electronic bistability in a monolayer (Fig. 3c). To investigate this point, we have irradiated the monolayer of **2** with a 904 nm laser diode at 10 K and then measured the temperature dependence of the XAS spectra up to 100 K (Fig. S12†). The coupled effect of the X-ray beam (SOXIESST effect,^28^ reported in Fig. S13†) and the laser light irradiation optically populates the hs-Co^II^ metastable state at 10 K, reaching a hs-Co^II^SQ percentage of 70(2)%. This value corresponds to a 19% overall conversion of the ls-Co^III^Cat phase present a 10 K, similarly to what is found for the powder sample (see Fig. S4†). Upon heating to 100 K, the hs-Co^II^SQ content decreases to 63(3)%, in line with a thermally activated relaxation of the photo-induced metastable phase to the thermodynamically more stable ls-Co^III^Cat. Direct comparison of the relaxation profiles for the monolayer and bulk phase of **2** points out that, in analogy to entropy driven interconversion, the possibility to optically trigger Valence Tautomerism of Co–dioxolene complexes is retained after their surface grafting, and that the energy barrier to thermally activated relaxation remains substantially unaffected by surface deposition.

## Conclusions

To conclude, this work provides the first evidence of thermal and optical control of the electronic state in a monolayer of switchable paramagnetic units anchored to a metallic substrate. To this aim, a chemical functionalisation protocol has been developed to couple the interconverting molecular core with a linker group able to covalently bind on gold surface. ToF-SIMS and XPS investigations clearly point out the retention of the bulk phase chemical and electronic structures after the deposition on surface for all the investigated systems and allow us to exclude the presence of physisorbed material. The XAS analyses of the thermally and optically driven interconversion processes in the monolayer evidence a quantitative transposition of the switchable capabilities from the bulk phase up to the surface deposit, even though the employed molecular system displays an incomplete conversion in the experimentally accessible temperature range. These results hold great promise for VT systems in the perspective of novel molecular-based devices that can be thermally and optically controlled.
