# Supplementary material for: Thermal and optical control of electronic states in a single layer of switchable paramagnetic molecules
Source: Chem Sci. 2015 Feb 12;6(4):2268–74. doi: 10.1039/c5sc00163c (PMC5645729; doi:10.1039/c5sc00163c)
Supplement: Supplementary file 1 [file SC-006-C5SC00163C-s001.pdf]

Electronic Supplementary Material (ESI) for Chemical Science.
This journal is © The Royal Society of Chemistry 2015

Thermal and optical control of electronic states in a single layer of switchable paramagnetic molecules

Electronic Supplementary Information (ESI)

Giordano Poneti,*[a,b], Lorenzo Poggini,[a] Matteo Mannini,*[a] Brunetto Cortigiani,[a] Lorenzo Sorace,[a] Edwige Otero,[c] Philippe Sainctavit,[d] Agnese Magnani,[e] Roberta Sessoli,[a] Andrea Dei[a]

[a]University of Florence, Department of Chemistry "Ugo Schiff" and INSTM Research Unit of Florence, via della Lastruccia 3-13, 50019 Sesto Fiorentino, Italy

[b]"Guglielmo Marconi" University, Department of Applied Science and Technology, via Plinio 44, 00193 Roma, Italy

[c]Synchrotron SOLEIL, BP 48 91192 Gif-Sur-Yvette, France

[d]Institut de Minéralogie, de Physique des Matériaux et de Cosmochimie, UMR7590 CNRS, Université Pierre et Marie Curie (Paris 6), 4 place Jussieu, 75252 Paris, France

[e] *University of Siena, Department of Biotechnologies, Chemistry and Pharmacy and INSTM Research Unit of Siena, Via A. Moro 2,, 53100 Siena, Italy*

email: giordano.poneti@unifi.it; matteo.mannini@unifi.it

Experimental Section

Bulk chemical characterization. Elemental analysis was carried out on a Carlo Erba EA1110 CHNS-O automatic analyser. ^{1}H-NMR spectra in $CDCl_3$ solution were recorded at 298 K on a 400 MHz Bruker Avance III spectrometer. UV−vis spectra of dichloromethane solutions were recorded on a Jasco V70 spectrophotometer using quartz cells with 1 cm optical path.

Bulk Magnetic measurements.

Magnetic characterization of polycrystalline samples was carried out with a Quantum Design MPMS superconducting quantum interference device (SQUID) magnetometer working in 1(10) kOe applied field in the 2 – 45 K (45 – 300 K) range. Photomagnetic data were acquired mixing the samples with KBr powder and pressing them into a pellet to ease light penetration in the whole material. Light coming from a 904 nm CW laser diode was coupled with an optic fiber inserted in the sample space through a hollow sample rod and collimated on the sample by means of an aspheric lens, yielding a radiant power on the sample of about 5 mW/cm^2. The reversibility of the process was checked. Magnetic moments were corrected for the diamagnetic contribution of the KBr, of the sample holder as well as with Pascal's constants. The measurement of the T_{LIESST} parameter involved monitoring the *hs*-Co^{II}SQ molar fraction after the photoinduction, warming the sample at 0.3 K/min up to complete relaxation.

Synthesis of DBCatSH ligand. 4,6-di-tert-butyl-3-((4-(mercaptomethyl)benzyl)thio)benzene-1,2-diol (DBCatSH) was prepared through minor changes of a previously reported route: a solution of 3,5-di-tert-butyl-quinone in hexane (22 mg, 0.1 mmol in 20 ml) was added to an excess solution of 1,4-benzen-dimethan-thiol (43 mg, 0.25 mmol in 20 ml of hexane). After the dark green solution turned colourless, the excess di-thiol was removed washing the hexane solution with an aqueous pH 12 buffer. The organic phase was thus dehydrated over $MgSO_4$ and evaporated to dryness, yielding DBCatSH in moderate yield (18 mg, 46%). Analysis (calcd, found for $C_{22}H_{30}O_2S_2$): C (67.65, 67.44), H (7.74, 7.75), S (16.42, 16.48). ^{1}H-NMR (400 MHz, 9.4 T, $CDCl_3$): δ 7.28 (d, J =8.3 Hz,

2H), 7.21 (d, J =8.3 Hz, 2H), 7.15 (s, 1H), 6.93 (s, 1H), 5.54 (s, 1H), 3.81 (s, 2H), 3.73 (d, J =7.6 Hz, 2H), 1.76 (t, J=7.6 Hz, 1H), 1.51 (s, 9H), 1.41 (s, 9H).

Synthesis of 1-3 complexes. Me_ntpa ligands synthesis followed previously established literature protocol.[1] **1-3** were synthesized adding trimethylamine deprotonated DBCatSH (78.0 mg, 0.2 mmol) to a disareated methanol solution of $CoCl_2 \cdot 6H_2O$ (47.4 mg, 0.2 mmol) and the proper methyl derivative of the tpa ligand (0.2 mmol). After gentle heating for 15 minutes, the solution was oxidized with dioxygen and the complexes were precipitated adding a water solution of KPF_6 (55 mg, 0.3 mmol in 5 ml H_2O) and recrystallized from a 1:2 mixture of dichloromethane and methanol. **1**: Yield: 135 mg, 74 %. Analysis (calcd, found for $C_{41}H_{50}CoF_6N_4O_3PS_2$): C (53.82, 53.98), H (5.51, 5.55), N (6.12, 6.10), S (7.01, 6.95). **2**: Yield: 130 mg, 69 %. Analysis (calcd, found for $C_{43}H_{54}CoF_6N_4O_3PS_2$): C (54.77, 54.84), H (5.77, 5.73), N (5.94, 5.91), S (6.80, 6.85). **3**: Yield: 124 mg, 65 %. Analysis (calcd, found for $C_{44}H_{56}CoF_6N_4O_3PS_2$): C (55.22, 55.38), H (5.90, 5.86), N (5.85, 5.91), S (6.70, 6.74).

Monolayer preparation. Monolayers of **1-3** complexes were prepared in a portable glove-bag by incubation (18 hours) in 2 mM dichlorometane solutions of the complexes of 150 nm thick Au(111) films evaporated on mica (flame annealed with hydrogen flame before the immersion); after incubation, the slides were rinsed several times with dichloromethane and dried with nitrogen to leave on the surface only the chemisorbed molecules.

ToF-SIMS characterization.

ToF-SIMS characterization was performed using a TRIFT III time-of-flight secondary ion mass spectrometer (Physical Electronics, Chanhassen, MN, USA) equipped with a gold liquid-metal primary ion source. Positive ion spectra were acquired with a pulsed, bunched 22 keV Au^+ primary ion beam, rastering the ion beam over a 10^4 μm^2 sample area. The primary ion dose was kept below 10^{11} ions/cm^2 to maintain static SIMS conditions.[2] Mass spectra were calibrated to CH_3^+ (m/z 15.023), $C_2H_3^+$ (m/z 27.023), $C_3H_5^+$ (m/z 41.039) and $AuC_2H_4^+$ (m/z 224.997) (the latter only for monolayer spectra). For the monolayer samples, mass resolutions (m/Δm) were 6000 for **1**, 5800 for

2 and 5600 for **3**, while for bulk ones they were 10000 for **1**, 13000 for **2** and 18000 for **3**. These variations in Δm/m do not alter significantly our analysis. All the samples were treated under inert atmosphere before the insertion in the ultra-high vacuum (UHV) chamber.

XPS characterization. XPS experiments were carried out in a UHV apparatus with a base pressure in the 10^{-10} mbar range. Monochromatised Al Kα radiation was used for XPS measurements (1486.6 eV, 100 W). The detector was an VSW hemispherical analyser mounting a 16-channel detector, the angle between the analyser axis and the X-ray source was 54.44° and the semicone angle of acceptance of the analyser was 4°. The XPS spectra were measured with a fixed pass energy of 44 eV. The binding energy (BE) scale was calibrated setting the Au *4f* signal of the substrate at 83.9 eV. In order to minimize air exposure and atmospheric contamination, samples were mounted on sample holder under dry nitrogen environment in a portable glove bag which was then connected to the fast-entry lock system of the XPS chamber. Spectral analysis consisted in a linear background subtraction and deconvolution using a mixed Gaussian and Lorentzian line-shapes for each spectral component. The stoichiometry of the samples was calculated by peak integration, using sensitivity factors reported for constant band-pass filter machines with similar geometric configuration in the www.uksaf.org database.

XAS characterization. XAS spectra have been acquired at the ID08 beamline of the European Synchrotron Radiation Facility (ESRF) in Grenoble, France. Drop cast samples have been prepared from 2 mM dichloromethane solutions. Monolayer and drop casted samples have been inserted into the cryomagnet working in the 8-300 K range and equipped with optical windows for sample irradiation with both an X-ray beam and a CW 904 nm laser beam. The photon flux per second of the 904 nm laser was 10 mW/cm^2 on the sample while the X-ray photon flux was reduced in order to avoid any radiation damage. The reduction was obtained by inserting horizontal and vertical baffles downstream the Apple II ID08 undulator and selecting 10 μm × 10 μm for the entrance and exits slits of the Dragon monochromator. Estimation of the temperature dependence of the *hs*-Co^{II} molar fraction of **2** was performed through least-squares interpolation of its normalized L_3 XAS

spectra with the ones of similar Cobalt-dioxolene complexes, featuring temperature independent *ls*-Co^{III}Cat and *hs*-Co^{II}SQ, recorded in similar experimental conditions.

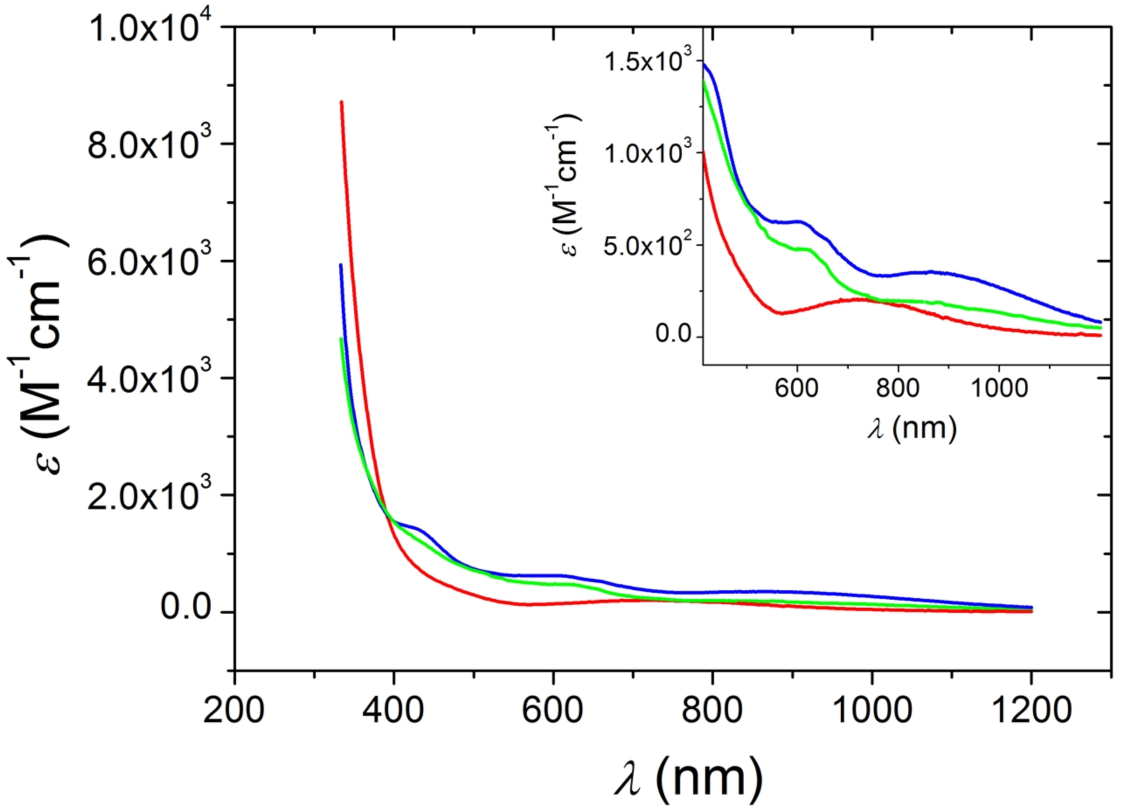

Figure S1. Room temperature UV-Vis absorption spectra of dichloromethane solutions of **1** (red line), **2** (green line) and **3** (blue line). *Inset*: magnification of the 450-1200 nm spectral region. The electronic spectrum of **1** displays the absorptions expected for low-spin-Co^{III} octahedral complexes coordinated to a catecholate ligand, showing a broad absorption centred at 740 nm, assigned to a ligand to metal electron transfer process, and a more intense band located in the UV region, related to π- π* internal transition of the dioxolene ligand.[3] The absorption bands found for **2** and **3** are related to internal transitions of the semiquinonate ligand (peaking at 430 and 880 nm) as well as charge transfer from the frontier orbitals of the metal to the π* orbitals of the ligand (500-700 nm), confirming the presence of a high-spin-Co^{II} ion bound to the semiquinonate radical form of the dioxolene ligand.[3]

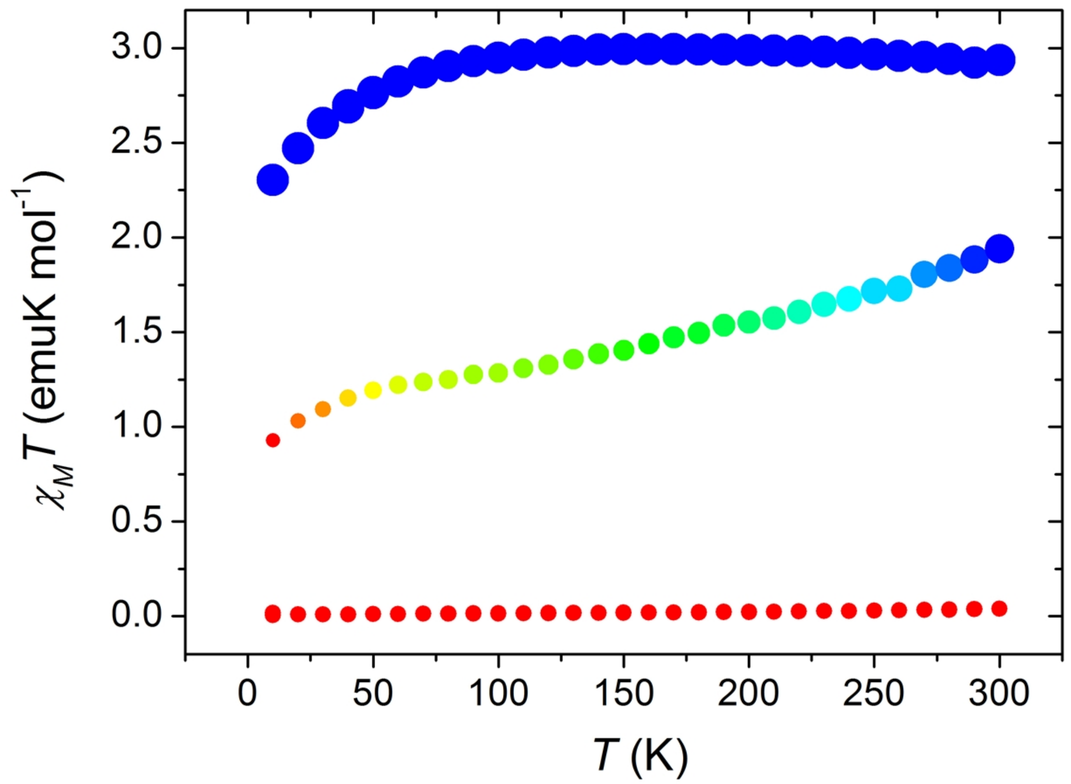

Figure S2. Temperature dependence of the $\chi_M T$ product of **1** (small red circles), **2** (changing full circles), and **3** (large full blue circles), measured applying a 1 kOe field in the 10 − 60 K range and a 10 kOe one up to 300 K. Size of circles is proportional to *hs*-Co^{II}SQ molar fraction.

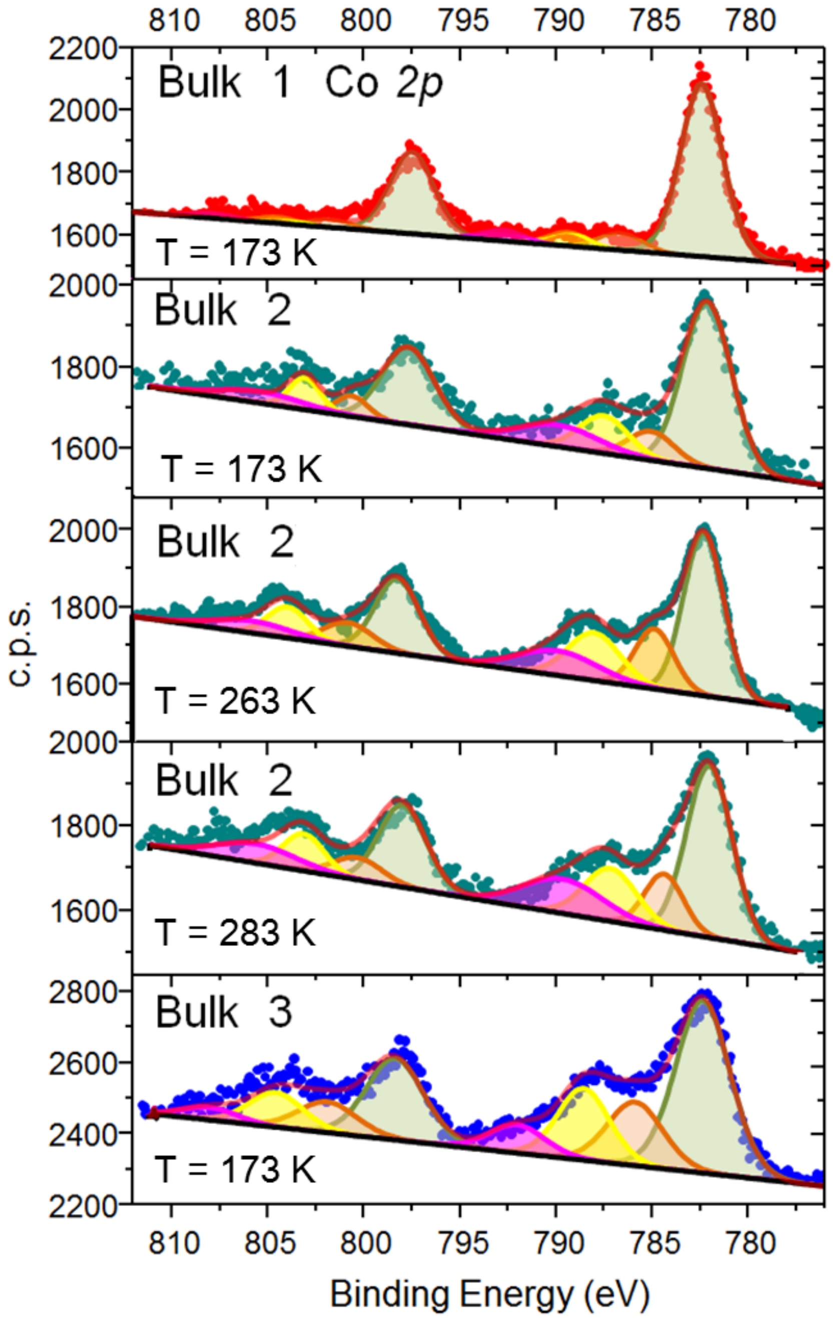

Figure S3. Bulk phase Co2*p* XPS spectra of compounds **1**−**3** along with best-fit components, as reported in Supplementary Table 2 (intensity is expressed in counts per second, cps).

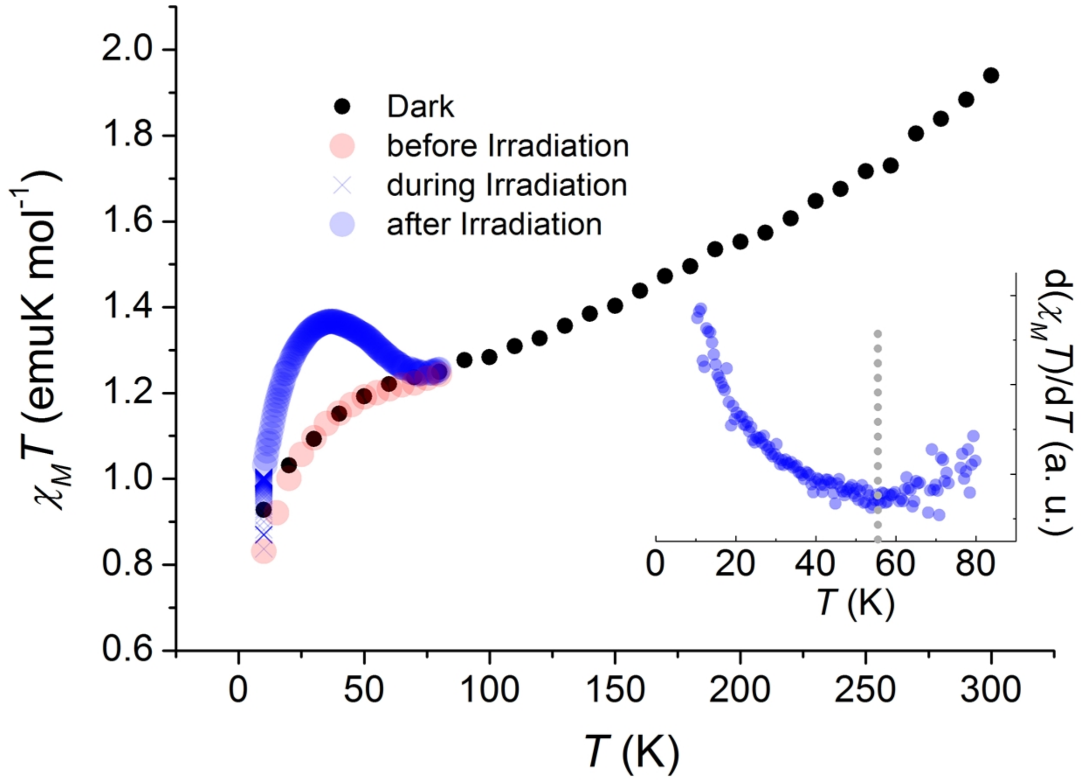

Figure S4. Temperature dependence of $\chi_M T$ of **2**, before (red circles), during (blue crosses) and after (blue circles) 904 nm irradiation at 10 K. The inset shows the derivative curve of the data recorded after irradiation, highlighting the T_{LIESST} value with the grey dotted line.

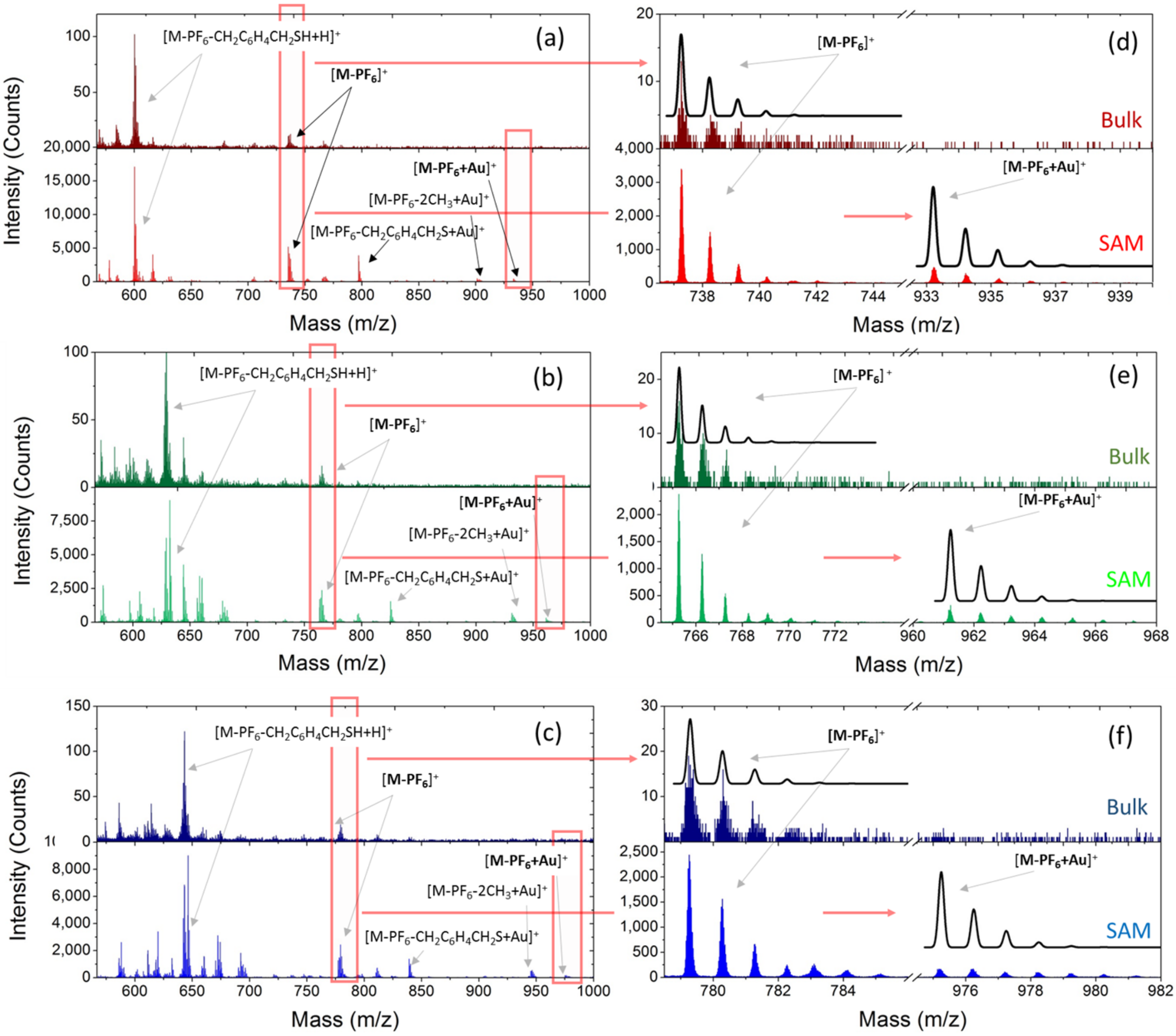

Figure S5. *Panels a-c)*: Positive ToF-SIMS spectra of bulk and SAMs of **1** (red sticks), **2** (green sticks), and **3** (blue sticks) measured in static regime, showing the fragmentation pattern in the 550 – 1000 m/z region. *Panels d-f)*: Magnification of the $[M\text{-}PF_6]^+$ and $[M\text{-}PF_6\text{-}H\text{+}Au]^+$ regions (enclosed in the red rectangles in a), b) and c)) for bulk and SAM samples of **1**-**3** complexes. The calculated isotopic distribution pattern expected for each fragment is reported as a black line.

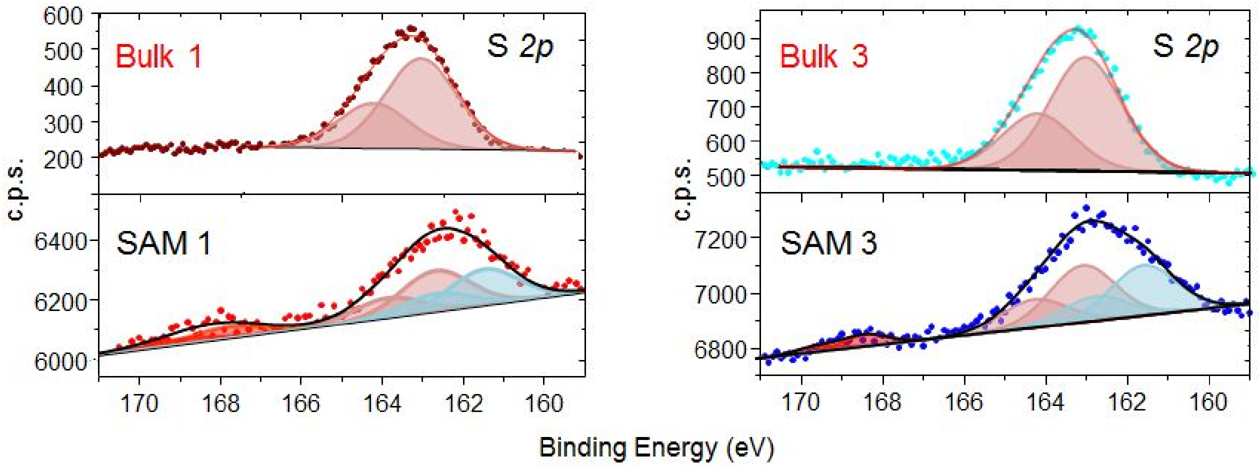

Figure S6. Bulk phase (above) and monolayer (below) S2*p* photoemission peaks of **1** (left) and **3** (right). Best fitting lines and contributions are reported. Intensity is expressed in counts per second (c.p.s.).

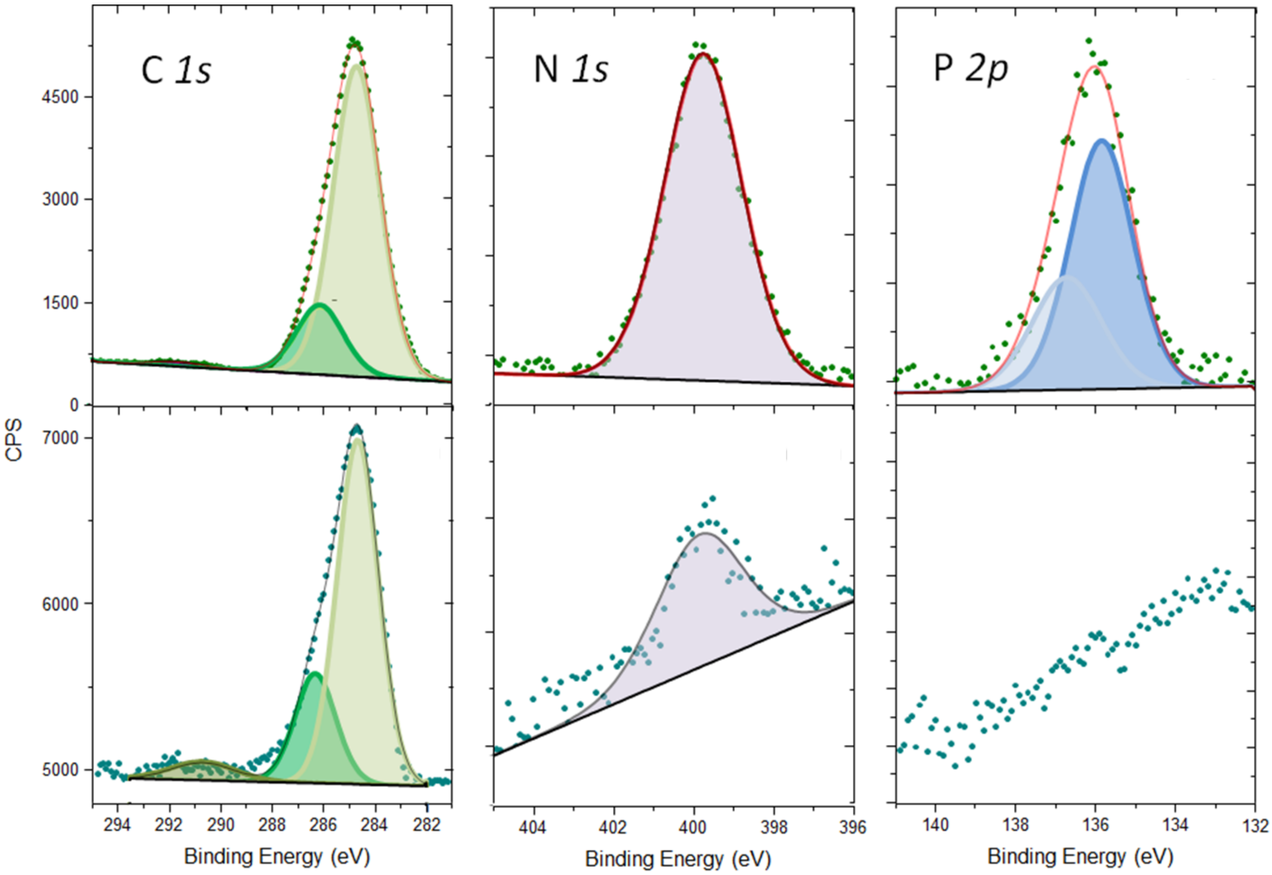

Figure S7. Comparison between bulk phase (above) and monolayer (below) room temperature XPS spectra of compound **2** along with best-fit components, as reported in Table 1. Intensity is expressed in counts per second (cps).

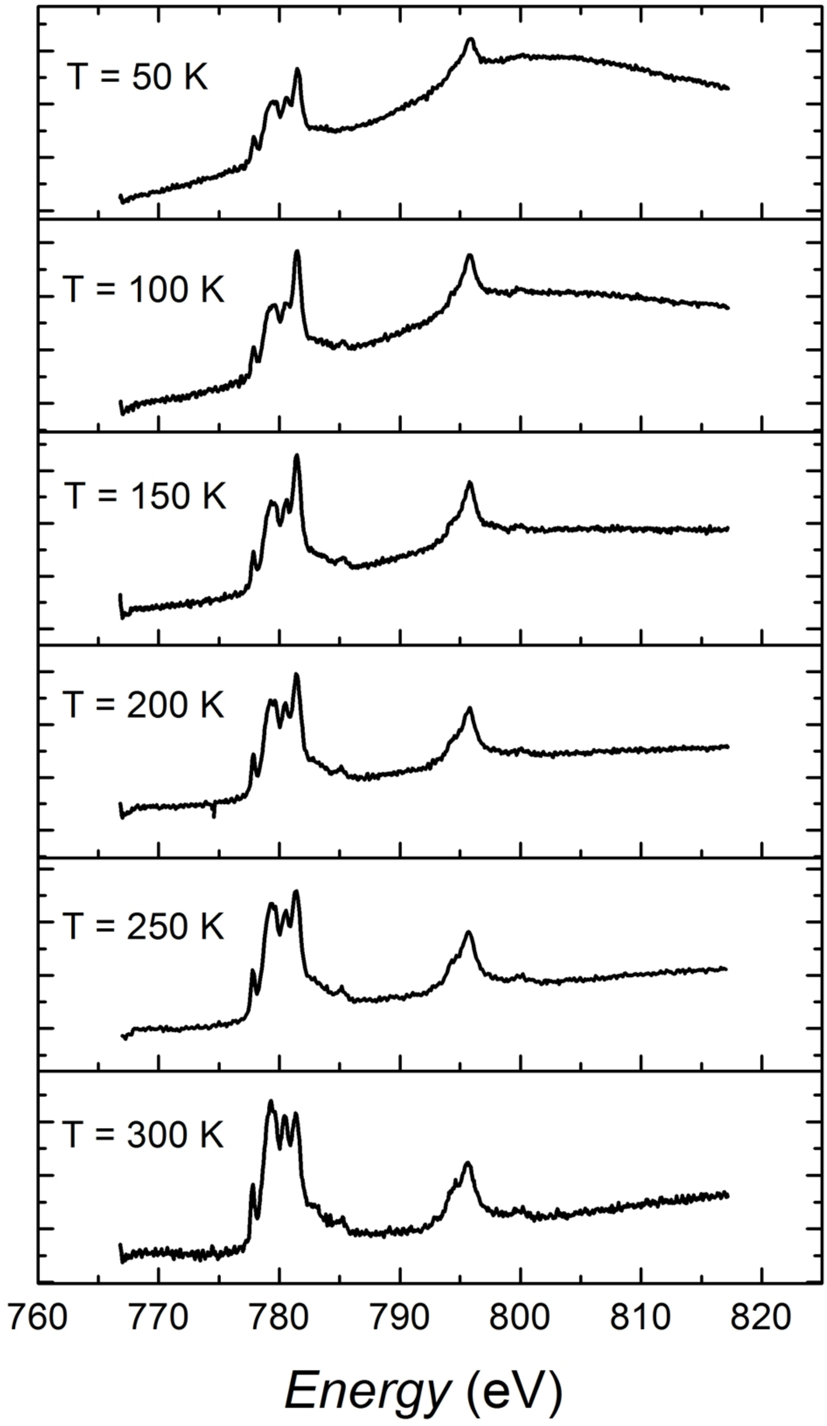

Figure S8. Thermal evolution of Co $L_{3,2}$ edges XAS spectra for a monolayer of **2**. As expected, the shape of background changes with temperature because of the appearance of Au XAFS oscillations when the temperature is lowered.

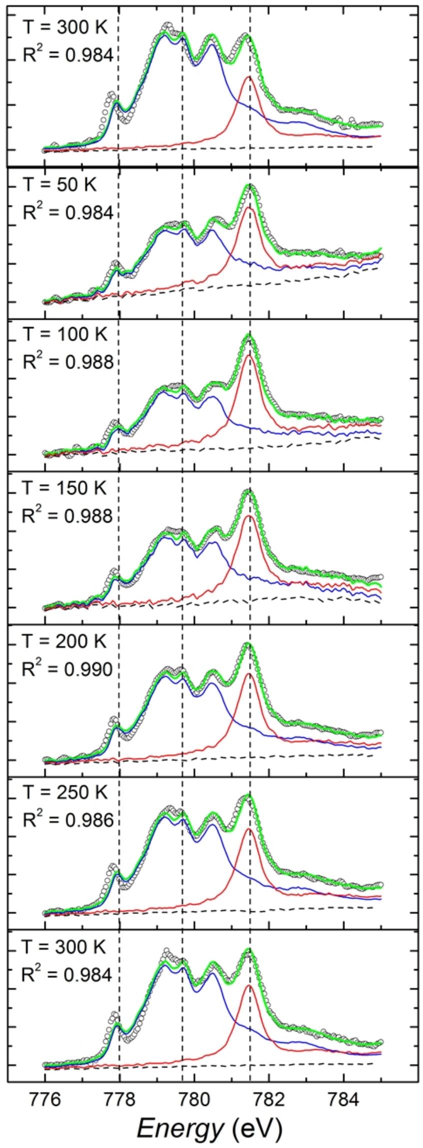

Figure S9. Temperature evolution of the 300 K Co L_3 edge XAS spectra of a monolayer of **2** (empty circles), along with best fitting lines (green line), calculated as described in the Methods section. High-spin Co^{II} and low-spin Co^{III} contributions and background are displayed as blue, red and broken black lines, respectively. The spectra, taken on the same spot of the sample, point out the occurrence of a reversible thermally driven high-spin Co^{II} to low-spin Co^{III} conversion on lowering the temperature, in line with an entropy driven Valence Tautomeric process at the monolayer level. Broken vertical lines correlate spectral features belonging to the different redox isomers throughout the series.

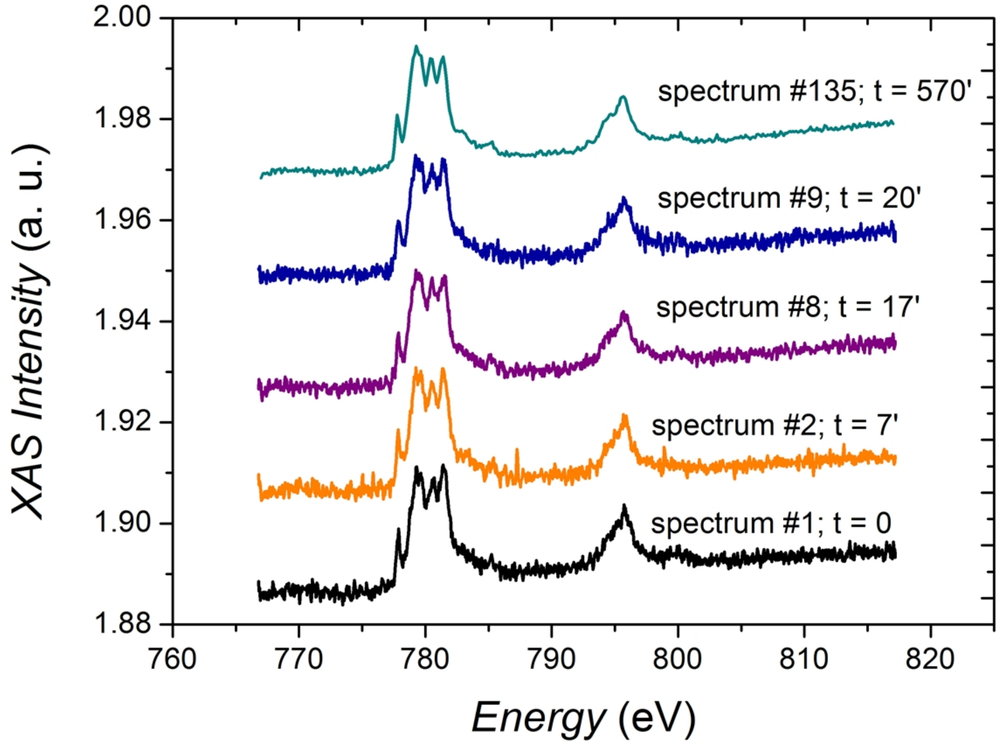

Figure S10. Isothermal time evolution of the XAS spectrum at 300 K for $L_{2,3}$ edges of a monolayer of **2**, measured on the same sample spot, excluding the occurrence of X-ray induced irreversible damaging of the system.

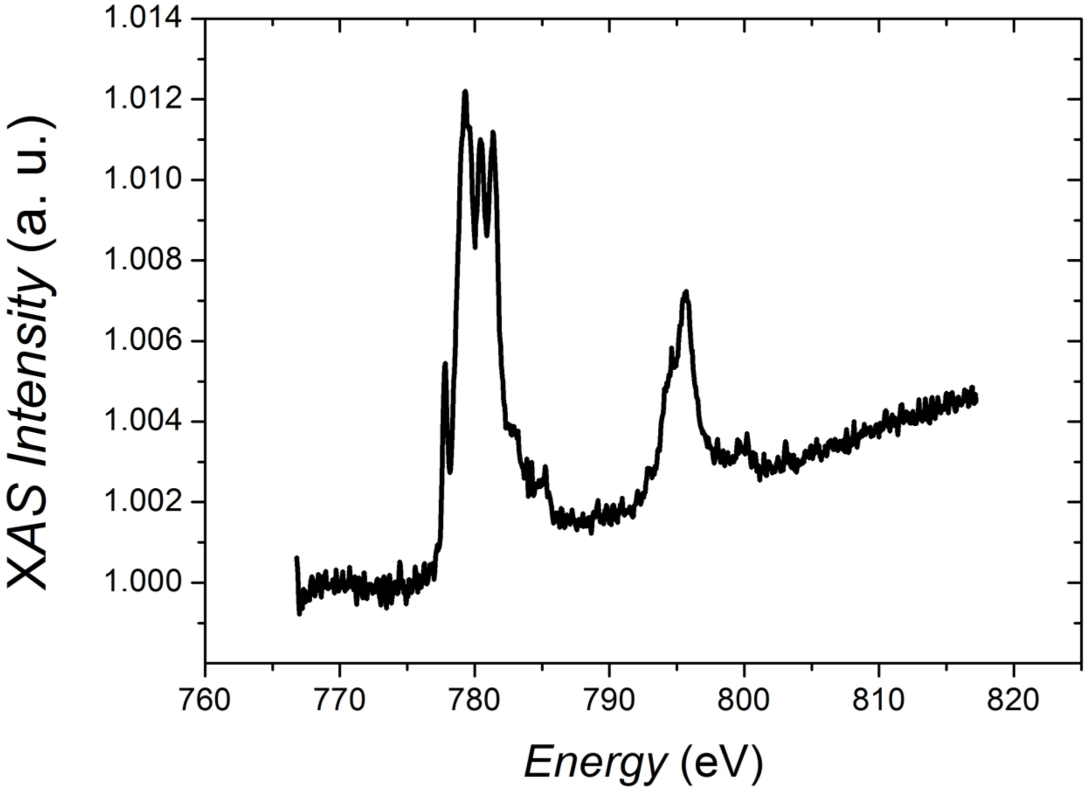

Figure S11. 300 K normalised Co $L_{2,3}$ spectrum of a monolayer of **2**, highlighting the 1.2 % absorption edge jump, in line with what expected for a monolayer of molecules.

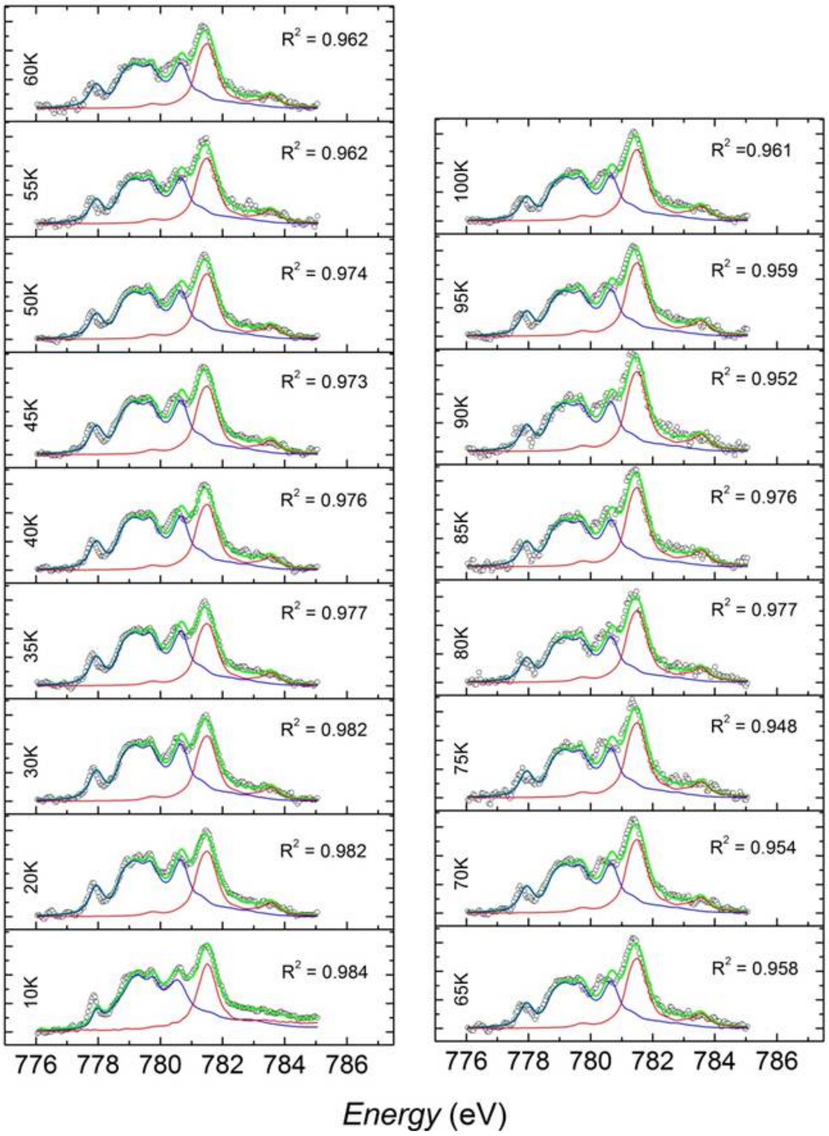

Figure S12. Thermal evolution of the normalised spectra of the Co L_3 edge of a monolayer of **2** measured after 904 nm laser irradiation, along with best fitting lines (green lines) and *hs*-Co^{II} and *ls*-Co^{III} fitting contributions (blue and red line, respectively).

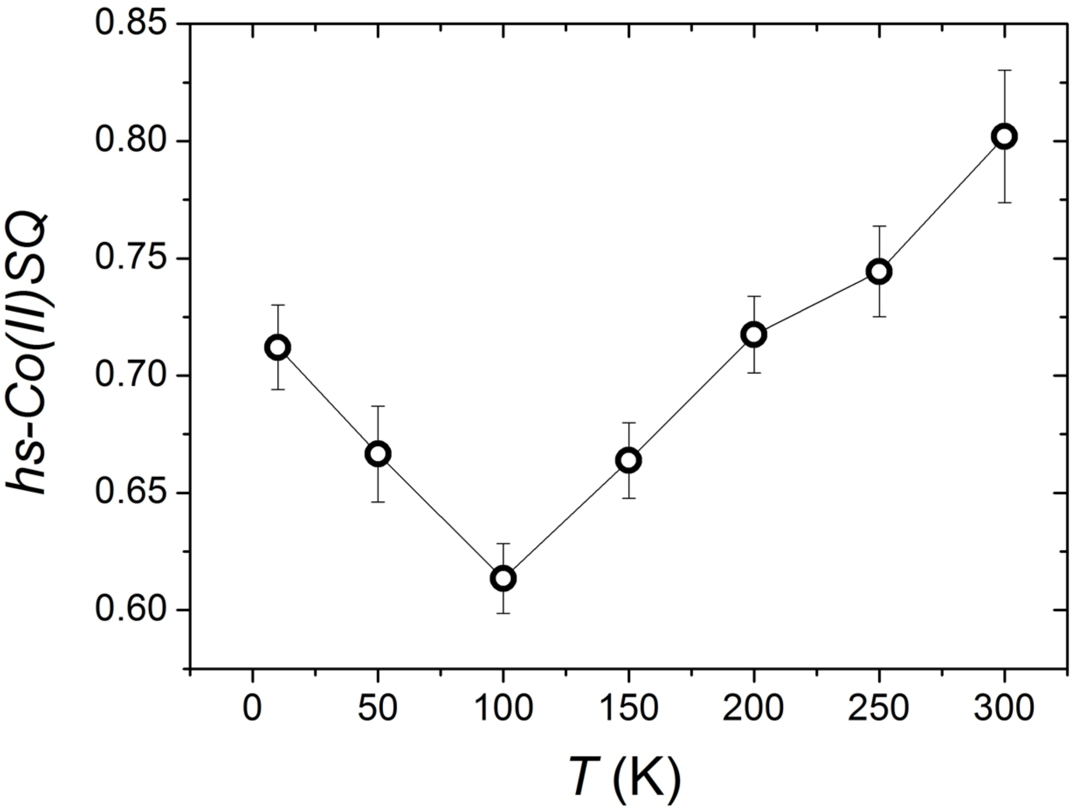

Figure S13. Temperature dependence of the high spin-Co(II)SQ molar fraction for a monolayer of **2** without laser light irradiation, pointing out the SOXIESST effect below 100 K.

Fragment	1					2					3				
	Theor. (m/z)	Bulk (m/z)		SAM (m/z)		Theor (m/z)	Bulk (m/z)		SAM (m/z)		Theor. (m/z)	Bulk (m/z)		SAM (m/z)	
$[M-PF_6+S-H+Au]^+$	965.17	n.d	-	965.15	(vw)	993.20	n.d	-	993.19	(vw)	1007.22	n.d	-	1007.19	(vw)
$[M-PF_6-CH_3+S-H+Au]^+$	950.14	n.d	-	950.16	(vw)	978.18	n.d	-	978.21	(vw)	992.19	n.d	-	992.20	(vw)
$[M-PF_6-H+Au]^+$	933.20	n.d	-	933.18	(w)	961.23	n.d	-	961.22	(w)	975.24	n.d	-	975.20	(w)
$[M-PF_6-CH_3+Au]^+$	918.17	n.d.	-	918.20	(vw)	948.22	n.d.	-	948.26	(vw)	961.23	n.d.	-	961.24	(vw)
$[M-PF_6-2*CH_3+Au]^+$	903.15	n.d.	-	903.20	(m)	931.18	n.d.	-	931.24	(m)	945.20	n.d.	-	945.25	(m)
$[M-PF_6-C(CH_3)_3+Au]^+$	8771.30	n.d.	-	nd	-	905.17	n.d.	-	905.13	(vw)	919.18	n.d.	-	919.14	(vw)
$[M-PF_6-C(CH_3)_3+Au-CH_3+2H]^+$	863.12	n.d.	-	863.12	(vw)	891.15	n.d.	-	891.15	(vw)	905.17	n.d.	-	905.16	(vw)
$[M-PF_6-CH_2C_6H_4CH_2S+Au]^+$	797.16	n.d.	-	797.15	(s)	825.19	n.d.	-	825.17	(m)	839.21	n.d.	-	839.16	(m)
$[M-PF_6-CH_2C_6H_4CH_2S-CH_3+H+Au]^+$	783.15	n.d.	-	783.18	(vw)	811.18	n.d.	-	811.23	(vw)	825.19	n.d.	-	825.20	(vw)
$[M-PF_6+S+H]^+$	769.21	n.d.	-	769.19	(vw)	797.24	797.25	(w)	797.23	(vw)	811.23	n.d.	-	811.23	(vw)
$[M-PF_6]^+$	737.24	737.25	(s)	737.22	(s)	765.27	765.26	(s)	765.25	(s)	779.29	779.29	(s)	779.26	(s)
$[M-PF_6-S+H]^+$	705.27	705.30	(w)	705.25	(vw)	733.30	733.29	(w)	733.28	(vw)	747.31	747.30	(w)	747.29	(vw)
$[M-PF_6-C(CH_3)_3+H]^+$	681.18	681.19	(vw)	681.00	(vw)	709.20	709.20	(vw)	709.20	(vw)	723.22	723.19	(vw)	723.16	(vw)
$[M-PF_6-CH_2C_5H_3NCH+H_3]^+$	646.20	n.d	-	n.d	-	660.21	660.38	(m)	660.37	(m)	674.22	674.35	(m)	674.36	(m)
$[M-PF_6-CH_3-CH_2C_5H_3NCH_3+2H]^+$	632.18	n.d	-	n.d	-	644.18	644.26	(m)	644.21	(m)	660.21	660.33	(w)	660.34	(m)
$[M-PF_6-CH_2C_5H_3N-2*CH_3+2H]^+$	616.15	616.23	(s)	616.18	(s)	632.18	632.31	(s)	632.34	(vs)	646.19	646.31	(s)	646.33	(vs)
$[M-PF_6-CH_2C_6H_4CH_2SH+H]^+$	601.20	601.23	(vs)	601.20	(vs)	629.24	629.25	(vs)	629.23	(s)	643.25	643.24	(vs)	643.22	(vs)
$[M-PF_6-CH_2C_5H_3N-CH_2S+H]^+$	600.21	600.22	(s)	600.19	(s)	612.21	612.20	(w)	612.19	(vw)	628.24	628.26	(m)	n.d.	-
$[M-PF_6-CH_2C_5H_3NCH_3-CH_3-CH_2S+H]^+$	586.20	586.17	(m)	586.20	(m)	600.21	600.24	(w)	n.d.	-	614.23	600.24	(m)	n.d.	-
$[M-PF_6-S-CH_2C_6H_4CH_2S+H]^+$	569.23	569.23	(m)	569.22	(m)	597.26	597.27	(m)	597.25	(m)	611.27	611.28	(m)	611.25	(m)

Table S1. Summary of ToF-SIMS peaks expected and experimentally found for bulk and SAMs samples of **1-3**.

Co *2p*

Bulk	Components A B.E. (%)	B B.E. (%)	C B.E. (%)	D B.E. (%)	ΔE_{SO}
(1)	782.4 (**76.2**)	786.7 (**8.2**)	789.4 (**7.4**)	792.8 (**8.3**)	**15.1**
(2), T= 173 K	782.1 (**62.2**)	785.0 (**10.3**)	787.5 (**14.2**)	789.9 (**13.4**)	**15.54**
(2), T= 263 K	782.3 (**47.1**)	784.9 (**16.7**)	788.0 (**17.8**)	789.9 (**18.4**)	**15.9**
(2), T= 283 K	782.0 (**50.3**)	784.4 (**14.0**)	787.2 (**17.9**)	789.7 (**17.8**)	**15.94**
(3)	782.3 (**48.0**)	785.9 (**19.2**)	788.5 (**18.9**)	792.0 (**13.8**)	**16.0**

SAM	B.E. (%)	B.E. (%)	B.E. (%)	B.E. (%)	ΔE_{SO}
(1)	781.5 (**50.9**)	784.3 (**20.1**)	788.3 (**14.7**)	792.5 (**14.4**)	**15.1**
(2), T= 173 K	781.4 (**46.5**)	785.2 (**18.6**)	787.3 (**10.2**)	789.8 (**24.8**)	**15.4**
(2), T= 263 K	781.2 (**37.6**)	785.0 (**26.1**)	787.8 (**12.7**)	790.2 (**23.6**)	**15.8**
(2), T= 283 K	781.0 (**40.5**)	784.8 (**24.4**)	787.6(**13.5**)	790.2 (**21.6**)	**15.8**
(3)	781.2 (**40.5**)	785.2 (**28.3**)	788.5 (**12.2**)	792.2 (**19.0**)	**16.0**

Table S2. Spectral components employed for the least squares fitting of the Co*2p* XPS photopeak of bulk (above) and monolayer (below) samples of **1-3**. Binding energies (B.E.) are in eV. In brackets are reported the integrated areas percentages of each component. ΔE_{SO} splittings are in eV.

$$\gamma_{hs-Co^{II}\ (SQ)}(T) = \frac{([\chi_M T\,(T)]_2 - [\chi_M T\,(T)]_1)}{([\chi_M T\,(T)]_3 - [\chi_M T\,(T)]_1)}$$

Equation S1: Expression used to quantitavely evaluate the high spin-Co^{II}SQ distribution profile of **2** using temperature dependent magnetometric studies. The $\chi_M T$ values of **1** and **3** as representative of low spin-Co^{III}Cat and high spin-Co^{II}SQ charge distributions, respectively.

Supporting References.

1. A. Beni, A. Dei, S. Laschi, M. Rizzitano and L. Sorace, *Chem. Eur. J.*, 2008, **14**, 1804-1813.
2. N. W. Ghonaim, M. Nieradko, L. Xi, H. Y. Nie, J. T. Francis, O. Grizzi, K. K. C. Yeung and L. W. M. Lau, *Appl. Surf. Sci.*, 2008, **255**, 1029-1032.
3. G. Poneti, M. Mannini, B. Cortigiani, L. Poggini, L. Sorace, E. Otero, P. Sainctavit, R. Sessoli and A. Dei, *Inorg. Chem.*, 2013, **52**, 11798-11805.
